# Nuclear, mitochondrial, and *Wolbachia* endosymbiont genomes of *Onchocerca lupi*, Portugal

**DOI:** 10.1128/msphere.00625-25

**Published:** 2026-01-26

**Authors:** Maria Stefania Latrofa, Ilenia Urso, Elisabetta Notario, Carmela Gissi, Carla Maia, Marinella Marzano, Graziano Pesole, Domenico Otranto

**Affiliations:** 1 Department of Veterinary Medicine, University of Bari Aldo Moro 9295, Bari, Italy; 2 Department of Biosciences, Biotechnology and Environment, University of Bari Aldo Moro 837102, Bari, Italy; 3 Institute of Biomembranes, Bioenergetics and Molecular Biotechnologies, National Research Council, Bari, Italy; 4 Global Health and Tropical Medicine, Associate Laboratory in Translation and Innovation Towards Global Health, Instituto de Higiene e Medicina Tropical, Universidade Nova de Lisboa, Lisboa, Portugal; 5 Consorzio Interuniversitario Biotecnologie 605849, Trieste, Italy; 6 Department of Veterinary Clinical Sciences, Jockey Club College of Veterinary Medicine and Life Sciences, City University of Hong Kong 597179, Hong Kong, Hong Kong; University of Wisconsin-Madison, Madison, Wisconsin, USA

**Keywords:** *Onchocerca lupi*, shotgun sequencing, nuclear genome, mitochondrial genome, *Wolbachia* endosymbiont genome, Portugal

## Abstract

Zoonotic *Onchocerca lupi* (Spirurida, Onchocercidae) has attracted the interest of the scientific community worldwide, by causing severe ocular infections in domestic animals (dogs, cats) and can infect wild carnivores (wolves, coyotes), as well as humans. Though recent advancements in scientific knowledge have been gained, gaps still remain about the biology of this filarioid, as well as its genetic structure. Based on mitochondrial genes, two highly divergent genotypes were identified, in the Iberian Peninsula (genotype 2) and Europe, Asia, and the United States (genotype 1), meanwhile only a draft nuclear genome of *O. lupi* from the United States is available. This study aimed to fill knowledge gaps about the genomic characterization of this filarioid and its *Wolbachia* endosymbiont. This study described the shotgun sequencing of an adult specimen of *O. lupi* isolated from a dog living in Portugal using the PacBio long-read sequencing technology. Three distinct genomes, such as the nuclear, mitochondrial, and *Wolbachia* endosymbiont, were assembled and analyzed. The assembled nuclear genome, Olupi_PT2024, exhibited high contiguity, accuracy, and completeness. Pairwise mitogenome comparative analyses among several *Onchocerca* species corroborated the high divergence between the two genotypes from Portugal and the USA, although the observed differences remained within the range of intra-species variation. The complete genome of the *Wolbachia* endosymbiont of *O. lup*i confirmed its classification within supergroup C and its close phylogenetic relationship with *Wolbachia* endosymbionts associated with the genus *Onchocerca*. The data on these three genomes may provide valuable resources for understanding the biology, population genetics, and phylogeography of this parasite.

**IMPORTANCE:**

*Onchocerca lupi*, a zoonotic parasite, causes ocular onchocerciasis in both domestic and wild carnivores, as well as humans. Despite recent scientific advances, gaps remain in both the biology and genetic structure of this parasite. To date, two genotypes have been described (genotype 1 distributed in Europe, Asia, and the United States, and genotype 2 circulating in the Iberian Peninsula) based on mitochondrial gene analysis. This study provided three distinct genomes (nuclear, mitochondrial, and *Wolbachia* endosymbiont) of *O. lupi* isolated from a dog living in Portugal. Overall, the data presented here corroborate the divergence between the two genotypes and provide new insights into the identification of genes that could serve as novel therapeutic targets for this filarial disease.

## INTRODUCTION

*Onchocerca lupi* (Spirurida, Onchocercidae) is a tissue-dwelling zoonotic filarial nematode, with a hitherto unknown arthropod vector (1–4). Over the past decade, *O. lupi* has attracted growing interest from the scientific community, being widely reported as a causative agent of ocular diseases in domestic animals (i.e., dogs and cats) (5–8) and can infect wild carnivores (i.e., wolves, coyotes) (9, 10) and humans (11). Indeed, from its first detection in a human patient from Turkey (12), the zoonotic potential of *O. lupi* has been strengthened by an increasing number of case reports worldwide (i.e., 22 cases spread in Europe, Asia, North Africa, and the United States) (11, 13). Despite its public health significance, knowledge gaps remain about its biology, population structure, phylogeography, and distribution (11), likely due to the absence of rapid and sensitive diagnostic tools to screen the animal’s infection (14).

To date, regardless of the imaging techniques available in alive animals (15), the diagnosis is still achieved by morphological (16) and molecular identification (17, 18) of adults embedded in ocular connective nodules (i.e., eyelids, conjunctiva, and sclera) and of subcutaneous microfilariae (mfs) isolated from skin biopsy (19). However, the latter diagnostic approach may lead to false-negative results, since mfs detection is highly dependent on their anatomical location, density, circadian rhythm, prepatent period, and/or previous microfilaricidal treatments (20). All of the above factors have contributed to an underestimation of the epidemiology of the nematode infection (21, 22). To overcome this gap, the performance of serological assays (i.e., Og4C3, DiroCHEK, SNAP Heartworm, and SNAP 4Dx Plus, indirect enzyme-linked immunosorbent assay) has also been assessed (14, 23, 24). However, the phylogenetic reconstructions of *O. lupi* based on cytochrome c oxidase subunit 1 (*cox1*) and NADH dehydrogenase subunit 5 (*nad5*) have highlighted the existence of two highly divergent genotypes in different geographic regions and animal hosts, with genotype 1 in dog, cat, and human hosts from USA, Canada, Europe, and Asia and genotype 2 in domestic animals from Spain and Portugal (25–27). To date, only two complete mitochondrial genomes ([28] Adema CM unpublished for OL964949) and one draft nuclear genome sequence of an adult nematode isolated from North American dogs are available (18).

The association of *Wolbachia* endosymbiont with *O. lupi* has been demonstrated in several studies, through the amplification of *Wolbachia*-specific genes from *O. lupi* specimens from Portugal (27) and Hungary (29). *Wolbachia* (order Rickettsiales) has a broad host range in arthropods and a more restricted distribution in onchocercids, as well as in a few plant-parasitic nematodes (30, 31). This endosymbiont exhibits a diverse lifestyle ranging from parasitism to obligate mutualism, depending on the host species (30, 32). The role of *Wolbachia* in the onchocercid (33) is associated with their life performances, such as molting, embryogenesis, growth, and in-host survival. Indeed, *Wolbachia* is localized in the female reproductive system and vertically transmitted to mfs through the egg cytoplasm during embryonic development (34).

This study aimed to fill the knowledge gap on the genetic data on *O. lupi* by applying a long-read shotgun sequencing strategy on an adult specimen isolated from a dog living in Portugal. Remarkably, this approach has allowed the assembly of the entire nuclear and mitochondrial (mtDNA) genomes of *O. lupi,* as well as the genome of its *Wolbachia* endosymbiont.

## MATERIALS AND METHODS

### Sampling and DNA extraction

An adult specimen of *O. lupi* (specimen ID: Olupi_PT2024) was isolated from the eye of an infected dog (3-year-old male), during a field study (35) conducted in the Algarve region, southern Portugal, an endemic area for the parasite (5). The adult worm contained within the host tissue nodule was preserved in ethanol 70% and transferred to the Department of Veterinary Medicine, University of Bari Aldo Moro. The nematode specimen was isolated under stereomicroscopy by removing the host’s tissue and subsequently washed four times with PBS. No other treatment was carried out (i.e., collagenase enzymatic digestion). Two fragments of the parasite were used for species confirmation by morphological analysis using keys described elsewhere (36) and by molecular identification using a quantitative real-time PCR (17). For the whole genome analysis, the genomic DNA was extracted from 33 mg of the Olupi_PT2024 sample using the NucleoSpin Tissue kit (Macherey-Nagel, Düren, Germany) according to the manufacturer’s recommendations. Fluorometric quantification and quality of the DNA were assessed with dsDNA HS assay for Qubit (ThermoFisher Scientific, Waltham, MA, USA) and Genomic DNA 165 kb Kit for Femto Pulse System (Agilent, Santa Clara, CA, USA), respectively. Based on the DNA amount obtained (approximately 400 ng) and the qualitative profile of the genomic DNA (peak at ~8.9 kb without significant smear below the peak, see Fig. S1), a PacBio (Pacific Biosciences) long-read sequencing approach was selected.

### Whole-genome library preparation and sequencing

A SMRTbell library (Pacific Biosciences, Menlo Park, CA, USA) was prepared using 11 ng of DNA and the SMRTbell prep kit 3.0, according to the Ultra-low DNA input protocol (unsupported protocol of May 2023, provided by PacBio). The Binding Kit 3.2, the Sequel II Sequencing Kit 2.0, and a single SMRT Cell 8M were used for sequencing on the PacBio Sequel IIe System.

### Long read analysis

Using bedtools v2.30.0, PacBio HiFi (High Fidelity) reads were extracted from the BAM files generated by the SMRT link v 13.1.0 and containing consensus sequences coming from the sequencing process. Before the assembly, reads were trimmed for adapter sequences using Cutadapt v4.2 (37). The genome size and heterozygosity were assessed by the *k*-mer-based statistical approach, and the *k*-mer profile was generated by reducing the sequencing data into substrings of length 32 using Meryl v1.3 (https://github.com/marbl/meryl). The information coming from *k*-mer frequency distribution was used as input for GenomeScope2.0 (38) to perform genome profile analysis.

### Contig-level assembly

Raw trimmed reads were assembled using Hifiasm v0.10.8-r525 (39) in HiFi-only assembly mode, setting k = 32 (*k*-mer length). The contiguity of the assembly, haplotypic duplications, and heterozygous overlaps were identified and resolved using purge-dups v1.2.6 (https://github.com/dfguan/purge_dups). The presence of putative contaminant sequences was evaluated with BlobTools v1.1 (40). The quality of the contig-level assembly was assessed through a series of quality metrics (i.e., total number of contigs, total contig length, N50, auN, L50, GC content %) using gfastats v1.3.8 (41) and summarized in a single snail plot using blobtool kit v4.4.4 command line (42). Assembly completeness was evaluated using Meryl v1.3 and Merqury v.1.3 (43), as well as with BUSCO v5.8.2 (Benchmarking Universal Single-Copy Orthologs) (44) using nematoda_odb10 as the benchmarking orthologs data set.

### *Wolbachia* genome assembly

The presence of the *Wolbachia* endosymbiont in *O. lupi* was revealed with BlobTools v1.1 by the identification of a subset of reads belonging to Pseudomonadota highly similar to the *Wolbachia* endosymbionts of both *Onchocerca ochengi* (HE660029.1) and *Onchocerca volvulus* (HG810405.1). *Wolbachia* HiFi reads were therefore filtered out by mapping them against a *Wolbachia* reference genome (HG810405.1) using minimap2 (v2.26), and the mapped reads were assembled with Flye (v2.9.5) (45) using the *–*meta option (special mode for metagenome assembly). The assembled contigs were checked for circularity using Flye’s “assembly_info.txt” output, where one circular contig was marked with a “Y” note. The Circlator (v1.5.5) (46) was used in circularization mode to confirm the genome circularity. The *Wolbachia* assembly quality was checked for basic statistics with gfastats (v1.3.8), and for completeness with BUSCO (v5.8.2) using the rickettsiales_odb10 database. The *wsp* gene was annotated by similarity with known *wsp* genes from other *Wolbachia* infecting onchocercid species.

### Genome annotation

A *de novo* repeat library was constructed using both RepeatModeler v2.0.3 with default parameters and the RepeatMasker v4.1.7 (47). The protein-coding prediction was assessed with an *ab initio* method using Augustus v3.5.0 software (48) with model parameters trained to the closely related genus *Caenorhabditis* (49–51).

### Mitochondrial genome assembly and annotation

The complete mitochondrial genome (mtDNA) of the *O. lupi* isolate collected from a dog from Portugal (Olupi_PT2024) was reconstructed using first MitoHiFi (52) and then Geneious Assembler (Geneious Prime 2025.0.3; https://www.geneious.com). Two different MitoHiFi runs were carried out, taking as primary input the Hifiasm contigs and the raw HiFi reads, respectively, and using the complete mtDNA of *O. lupi* from New Mexico (accession number: OL964949) as a reference. The seven identified mt-like contigs, corresponding to partially overlapping regions of the mtDNA with different lengths of several homopolymeric A and T stretches, were then assembled with the Geneious Assembler (Geneious Prime 2025.0.3; https://www.geneious.com), using the above mtDNA as reference. The final contig was inspected for mtDNA completeness, and its accuracy was verified by mapping on it the original HiFi reads with Minimap2 v2.26. The mtDNA of Olupi_PT2024 was annotated by comparison with the 17 complete mtDNAs of four *Onchocerca* and three *Dirofilaria* species available in the NCBI “nucleotide (nt)” database in Jan 2025 (https://www.ncbi.nlm.nih.gov/nucleotide/), including the two mtDNAs of *O. lupi* genotype 1 belonging to different populations (one sampled in Arizona in 2010 and the other in New Mexico in 2017; Table S1). As a prerequisite to the annotation of the Olupi_PT2024 mtDNA, the gene order and content of the 17 mtDNAs were checked and, if needed, corrected according to the curated mtDNA annotations of the nine Filarioidea species available in MitoZoa, a curated database of complete/nearly complete mtDNAs of Metazoa (53). Mitochondrial tRNA annotations of Olupi_PT2024 were also verified using Arwen (54). All mtDNA/gene alignments and annotations were performed with the tools present in Geneious Prime 2025.0.3 (https://www.geneious.com) and visually inspected and optimized. The intra-species nucleotide divergence was investigated in the *Onchocerca* species (*O. flexuosa, O. lupi, O. ochengi,* and *O. volvulus*) for which complete mtDNAs were available for at least two specimens. Sampling locality and date of these *Onchocerca* specimens were listed in Table S1. Intra-species nucleotide divergences were calculated as pairwise PAUP* uncorrected distances (i.e., percentage of sequence differences) on the entire mtDNA, as well as on the different gene categories (protein-coding and rRNA genes), the longest non-coding region (l-NCR), the first plus second codon positions (P12), and the third codon position (P3) (55).

### Phylogenetic analyses of *cox1* and *wsp*

The analyzed *cox1* data set consists of the longest sequences of the *Onchocerca* and *Dirofilaria* species that show the highest similarity to that of Olupi_PT2024. These sequences were selected through a Blastn search (56) (https://blast.ncbi.nlm.nih.gov/Blast.cgi) against the NCBI “nt” database (https://www.ncbi.nlm.nih.gov/nucleotide/, Jan 2025), using as query the previously annotated *cox1* of Olupi_PT2024, and limiting the search to a given range of genera and sequence length by using the Entrez query syntax. Identical or almost identical *cox1* sequences, corresponding to the same haplotype and identified during the subsequent alignment step (see below), were removed from the final *cox1* data set (see Table S1). Two *Dirofilaria* species were used as outgroups to root the reconstructed phylogenetic trees.

The analyzed *wsp* data set included representative sequences from valid *Wolbachia* supergroups as defined by a multilocus sequence typing approach (MLST) (30, 32, 57, 58). The MLST-unsupported supergroup G, proposed based solely on *wsp* inference and then considered an artifact resulting from extensive *wsp* recombination (57), was excluded. The supergroups infecting only onchocercids (C, D, and J), the supergroup infecting both onchocercids and arthropods (F), a few arthropod-restricted supergroups (A, B), and the Collembola-specific supergroup (E) were analyzed. Literature review combined with a Blastn search against the NCBI “nt” database, using as a query the annotated *wsp* of Olupi_PT2024, was performed for the retrieval of representative sequences of these supergroups. After removing identical or highly similar haplotypes, a single representative *wsp* sequence per onchocercid species was retained in the final data set (Table S2). For supergroups A and B, the few analyzed representative sequences were selected from those previously reported in reference 59.

Each sequence data set was translated and aligned at the amino acid (aa) level with Seaview v5.0.5 (60). The aa alignment was manually optimized, reverse translated to the corresponding nucleotide (nt) alignment, and used for phylogenetic reconstructions. The final *cox1* alignments, named “longest_cox1,” consisted of a total of 1,653 nt positions with 1,061 gapped sites*,* as it included both complete and partial *cox1* sequences. The *cox1* region of this alignment, commonly used as a DNA barcode for species identification, was saved in the alignment named “barcode_cox1,” which consisted of 694 nt positions with 69 gapped sites. After manual identification and removal of the poorly aligned 5′ and 3′ regions, a final *wsp* alignment of 510 nt positions was obtained.

Phylogenetic trees were reconstructed with the maximum likelihood (ML) method using the PhyML-SMS v3.0 online software, which included the automatic model selection algorithm Smart Model Selection (SMS) (61, 62) (http://www.atgc-montpellier.fr/phyml-sms/). Branch supports, indicating node reliability, were calculated by standard non-parametric bootstrap analysis based on 1,000 replicates. For *cox1* alignments, the best-fit substitution model selected under the Akaike Information Criterion (AIC) was the GTR+I for both the “longest_cox1” and the “barcode_cox1” alignments, so the trees were inferred under the model GTR+I+F. The proportion of invariant sites (I) was estimated by the PHYML v3.0 software itself. For *wsp*, the best-fit substitution model selected under AIC was the GTR+I+G, with the proportion of invariant sites estimated by the PHYML v3.0 software itself.

## RESULTS

### The nuclear genome Olupi_PT2024

A total of 2.5 million PacBio HiFi trimmed sequences were generated, with an average read length of ~3,6 kb and a max length of ~19 kb. Based on *k*-mer counting performed on the HiFi reads, the estimated genome size was ~92 Mbp, with a heterozygosity of 0.2% and 4% of repeated sequences. The mean *k*-mer coverage for the heterozygous (diploid) peak was located at 46.2×, while the homozygous peak (haploid) was at ~92× (Fig. S2).

The estimated genome size was consistent with that of Olupi_Ro2020_NM (18), with an initial contig-level assembly of ~102 Mbp, with 584 total contigs, an N50 value of ~615 kb, and a sequencing coverage of ~100×. This assembly was then purged from haplotigs and heterozygous overlaps, resulting in a total of 308 contigs, with an N50 value of ~670 kb and 97.4% complete BUSCO genes within nematoda_odb10.

Results from contamination analysis revealed the presence of a fraction of contigs, putatively belonging to Pseudomonadota, Arthropoda, and Chordata phyla, which were removed from the assembled Olupi_PT2024 genome. The BUSCO gene content was then recalculated at each round. The removal of contigs classified as Chordata and Arthropoda caused a drop in the BUSCO “complete Nematoda genes” category, from 97.4% to 79.5% and 63%, respectively, suggesting that those contigs were more likely to belong to the Nematoda phylum rather than to actual contaminations from other species. On the contrary, the BUSCO “complete Nematoda gene” content was weakly affected by the removal of all contigs classified by BlobTools as Pseudomonadota (change from 97.4% to 95.2%). Therefore, a total of 13 contigs classified as Pseudomonadota were removed from the final assembly, while all contigs classified as Arthropoda and Chordata were retained. These 13 contigs matched two sequences of *Wolbachia,* providing proof of the *Wolbachia* presence.

After purging and decontamination, Olupi_PT2024 assembly comprised 295 contigs with a total length of ~92 Mbp and an N50 length of ~670 kb (Table 1).

**TABLE 1:** Genome assembly statistics of Olupi_PT2024 (genotype 2) after purging and decontamination, and of the Olupi_Ro2020_NM (genotype 1 [18]) genome assembly calculated by gfastats

Parameter	Olupi_PT2024	Olupi_Ro2020_NM
Total length	92,481,251	92,491,485
GC content (%)	29.14	29.16
# contigs	295	2,320
Largest contig	2,235,396	531,805
Contig N50	670,258	96,493
Contig L50	46	276
Contig auN	740,568.62	126,272.35
BUSCO complete single copy (%)	93.7	94.9
BUSCO complete duplicate (%)	1.5	0.5
BUSCO fragmented (%)	0.5	1.7
BUSCO missing (%)	4.3	2.9

**Fig 1:**
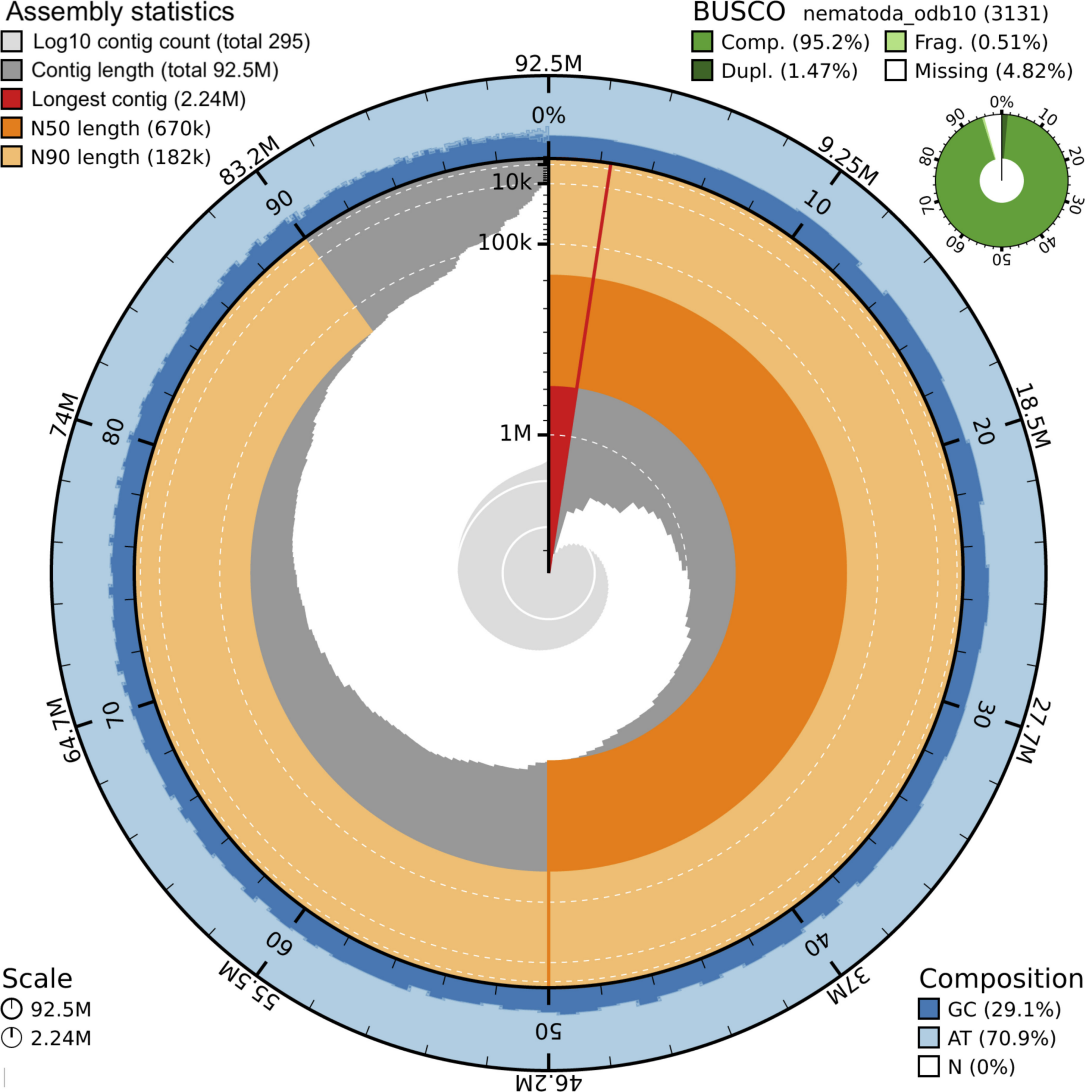
BlobToolKit snail plot showing the Olupi_PT2024 assembly metrics.

A graphic visualization of the most relevant Olupi_PT2024 assembly metrics was reported in Fig. 1.

Assembly quality assessment, according to universal single-copy orthologs, displayed 93.7% complete single-copy BUSCO genes in the Nematoda lineage, 1.5% as complete duplicated, 0.5% as fragmented and 4.3% as missing BUSCO genes (Table 1; Fig. 1). The comparison of the main assembly statistics from Olupi_PT2024, genotype 2, to those of the Olupi_Ro2020_NM assembly, genotype 1 (https://www.ncbi.nlm.nih.gov/datasets/genome/GCA_028564675.1/) (18) was reported in Table 1. The two genome assemblies have almost identical length (92,481,251 Mb vs 92,491,485 Mb), GC percentage (29.14% vs 29.16%), and BUSCO values, while differing greatly in the total contig number and other contig parameters. Analysis of the base-level accuracy and completeness performed with Merqury produced a quality value (QV) of 43.07, indicating a consensus accuracy >99.99% (QV: log-scaled probability of error for the consensus base calls [43]) and a *k*-mer completeness of 93.59% (completeness: fraction of reliable *k*-mers in the read set found in the assembly [43]) (Table 2). Both these values were indicative of a high-quality assembly.

**TABLE 2:** Consensus QV and *k*-mer completeness statistics of Olupi_PT2024 calculated by Merqury^*a*^^,^^*b*^

Parameter	Olupi_PT2024
Assembly only	145,725
Total *k*-mers	92,472,106
QV score	43.07
*k*-mer assembly	86,325,068
*k*-mer reads	92,239,507
Completeness	93.59%

^*a*^ QV score was calculated assuming that all *k*-mers in the assembly should be found at least once in the read set, thus estimating the probability that a base in the assembly was correct. *k*-mer completeness values were calculated as the fraction of reliable *k*-mers in the read set that were also found in the assembly.

^*b*^ “Assembly only”: unique *k*-mers which were present only in the assembly; “Total *k*-mers”: total number of unique *k*-mers present in both assembly and the read set; “*k*-mer assembly”: total number of unique *k*-mers which were present in the assembly; “*k*-mer reads”: unique *k*-mers that can be found in the input reads; “% completeness”: percentage of *k*-mers in the assembly relative to the total *k*-mers in the reads set.

The assembly (Fig. S3A) and copy number (Fig. S3B) spectrum plots showed the high quality of the Olupi_PT2024 assembly, with a large portion of the target genome correctly reconstructed and a read coverage of ~100×.

The annotation of the repeat elements showed that 10.69% of the genome was represented by repetitive elements, with 4.08% of them consisting of interspersed repeats (Table 3). The *ab initio* gene prediction identified a total of 12,086 coding genes (Table 3), comparable with those estimated for other *Onchocerca* species (range: 12,109–16,119 for *O. flexuosa, O. ochengi,* and *O. volvulus*), according to WormBase; https://parasite.wormbase.org/Onchocerca_flexuosa_prjeb512/Info/Index; https://parasite.wormbase.org/Onchocerca_ochengi_prjeb1204/Info/Index; https://parasite.wormbase.org/Onchocerca_volvulus_prjeb513/Info/Index/).

**TABLE 3:** RepeatMasker output for Olupi_PT2024 genome, detailing the percentages of identified repeats among specific classes, together with *ab initio* gene prediction estimated by the Augustus v3.5.0 software

Parameter	Number	Total length (bp)	% of the assembly	Mean size (bp)
Retroelements	1,658	1,213,064	1.31	
Rolling circles	9,256	2,457,497	2.66	
Unclassified	15,660	2,559,609	2.77	
Total interspersed repeats		3,772,673	4.08	
Small RNA	466	92,300	0.1	
Simple repeats	63,601	2,803,086	3.03	
Low complexity	15,170	760,559	0.82	
Total masked		9,886,115	10.69	
Protein-coding gene	12,086			3,783
Exon	91,735			192
5′ UTR	12,032			136
3′ UTR	12,059			207

### *Wolbachia* assembly and classification

A total of 19,499 HiFi reads identified as derived from *Wolbachia* DNA were used to assemble the endosymbiont genome of Olupi_PT2024 by a *de novo* approach. The Flye assembler identified a circular contig, confirmed also by Circlator.

**Fig 2:**
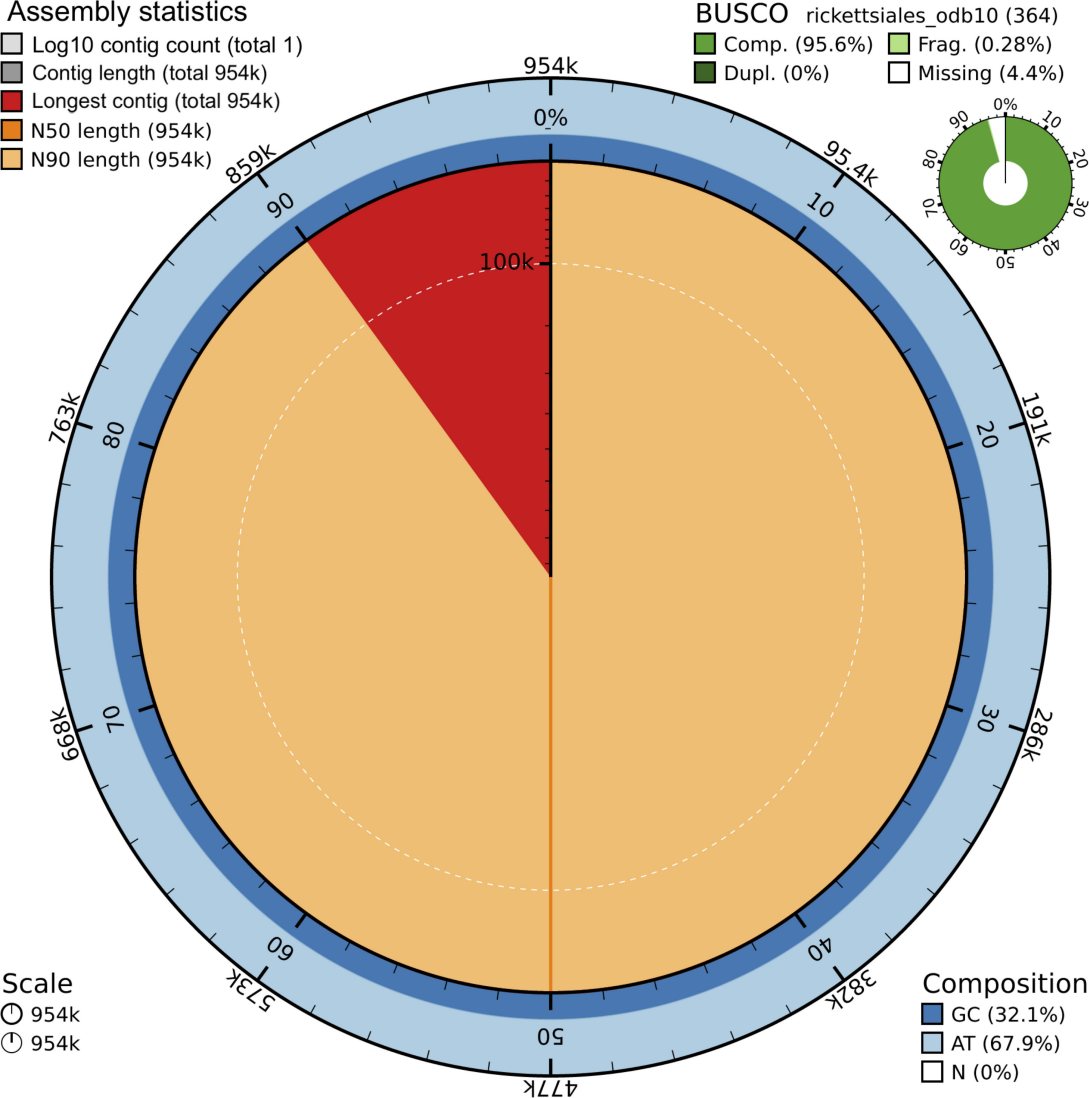
BlobToolKit snail plot showing the Olupi_PT2024 *Wolbachia* endosymbiont assembly metrics.

The final *Wolbachia* assembly, named wb_Olupi_PT2024, exhibited a 127× coverage and comprised a single contig with a total length of ~954 kb. The BUSCO analysis showed that 95.6% of the expected conserved *Rickettsiales* genes were complete and single copy, without duplicated genes, while 0.3% were fragmented and 4.1% were missing genes (Fig. 2).

The genome size of wb_Olupi_PT2024 was consistent with that of other *Wolbachia* genomes sequenced from *Onchocerca* and *Dirofilaria* species, which range from 920 to 998 kb. The *Wolbachia* assembly showed high completeness according to BUSCO, comparable to the completeness values (96%–99%) reported for related genomes, as estimated by the NCBI CheckM analysis (Table S3).

**Fig 3:**
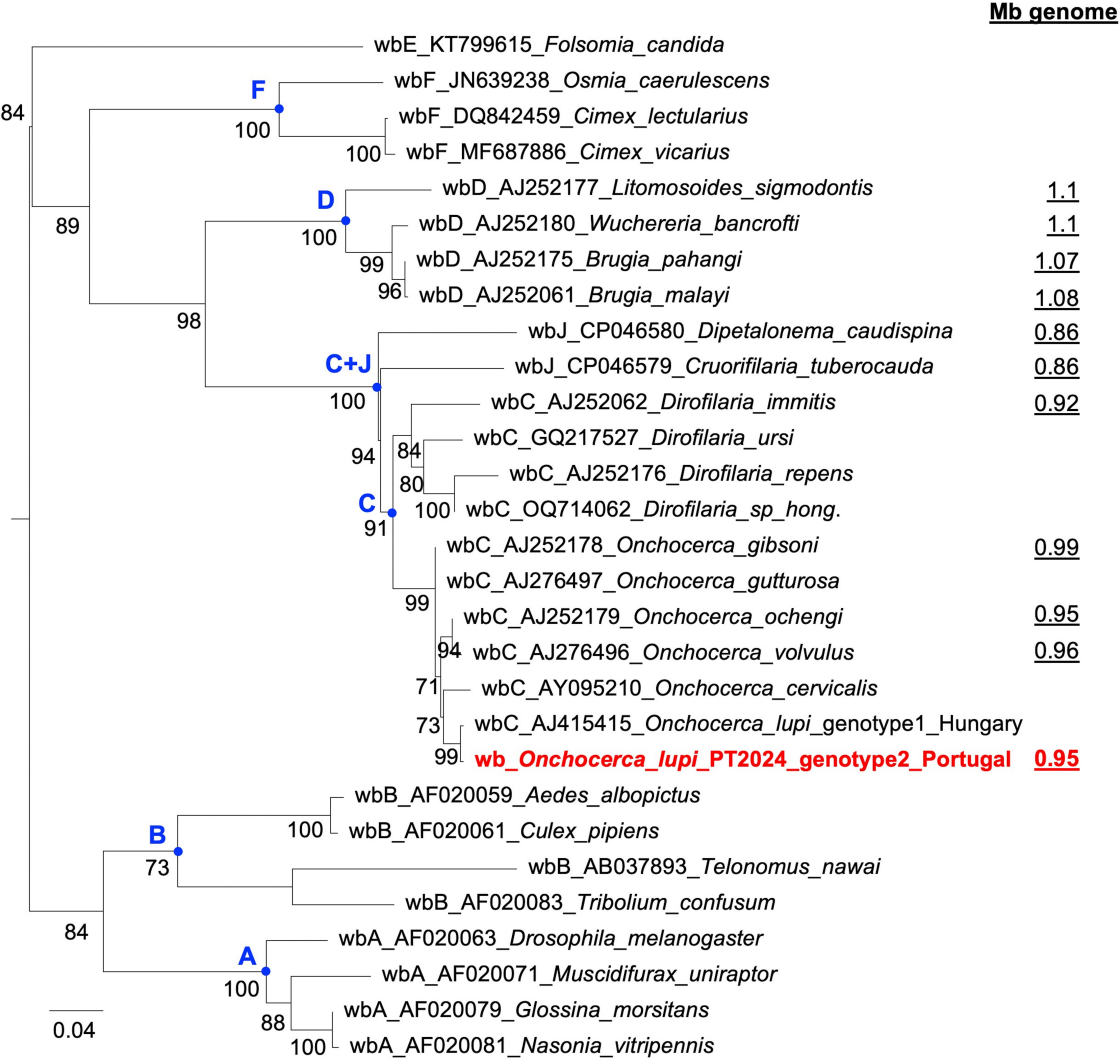
ML tree of *wsp* sequences from seven representative *Wolbachia* (wb) supergroups, together with the available genome size of *Wolbachia* strains from Onchocercidae. ML bootstrap values, based on 1,000 replicates, are shown only when ≥70%. *Wolbachia* genome sizes are underlined. Sequence names consist of the following parts, separated by underscores: *Wolbachia* (wb) supergroup, AC number of the *wsp* sequence, and host species of *Wolbachia*. The *wsp* from Olupi_PT2024 is highlighted in red. Nodes representing supergroups are shown in blue. Sampling localities are provided only for *O. lupi*. The tree is midpoint-rooted for clarity. Metadata for the reported *wsp* sequences and *Wolbachia* genomes are provided in Tables S1 and S3, respectively.

The *wsp* of wb_Olupi_PT2024 differs by two synonymous nucleotide substitutions from that of the genotype 1 *O. lupi* specimen from Hungary (29). The *wsp* phylogenetic reconstruction (Fig. 3) clustered wb_Olupi*_*PT2024 with all other *Wolbachia* from *Onchocerca* and indicated that it belongs to supergroup C with a high bootstrap support (91%). Indeed, all *Wolbachia* from *Onchocerca* species constitute a strongly supported clade (bootstrap 99%), as that from *Dirofilaria* species (bootstrap 84%), and form a well-supported clade with supergroup J (bootstrap 100%). Moreover, a robust sister-group relationship (bootstrap 98%) was observed between the supergroups infecting onchocercid hosts (i.e., D, C, and J). The supergroup infecting both onchocercids and arthropods (F), for which only arthropod *wsp* sequences were available, was also strongly supported (bootstrap 100%) (Fig. 3).

### Phylogenetic analysis of *cox1*

**Fig 4:**
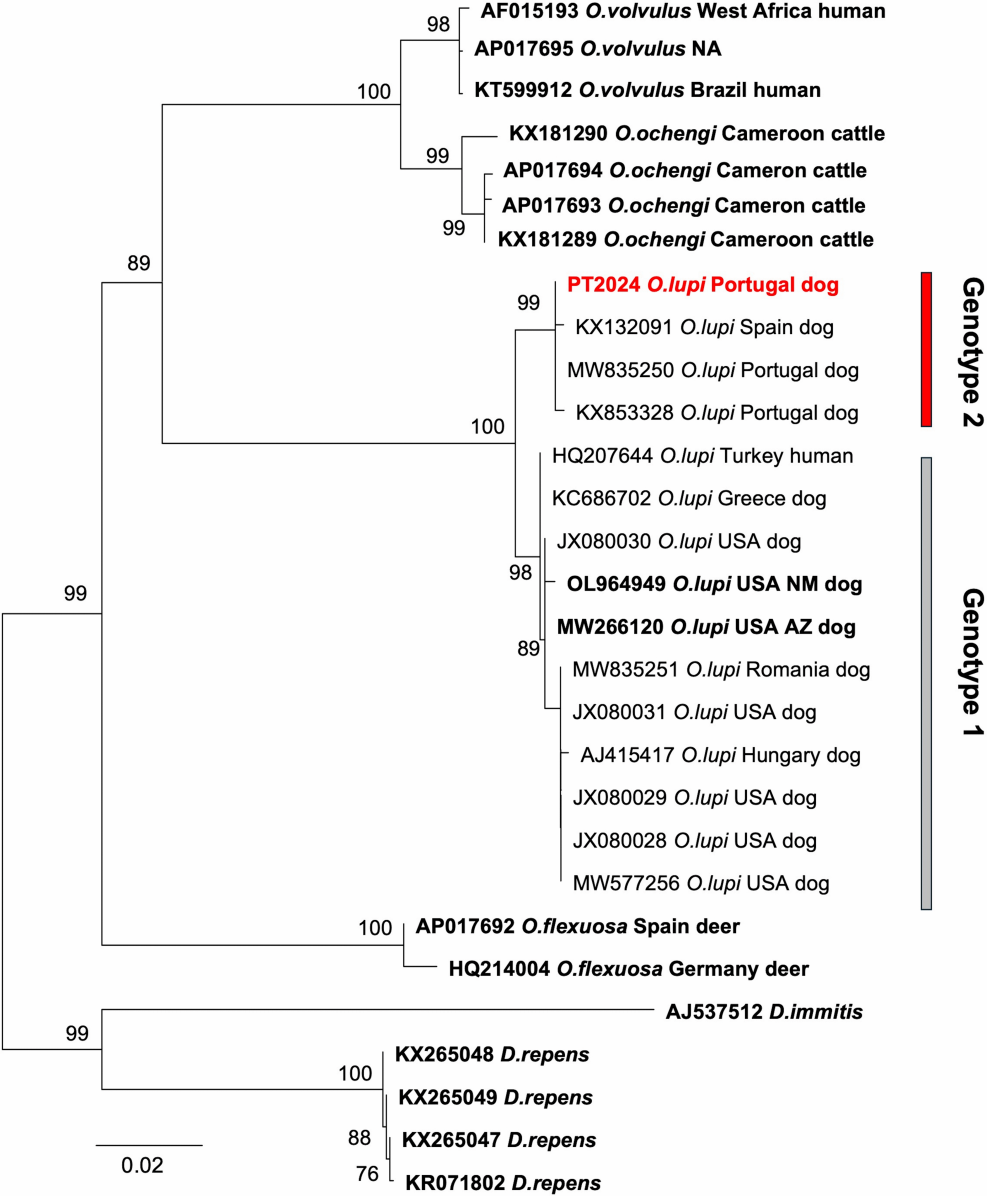
ML tree of *cox1* reconstructed from the “longest_cox1” alignment (1,653 nt positions). ML bootstrap values, based on 1,000 replicates, are shown only when ≥70%. *Dirofilaria* sequences were used as outgroups. The *cox1* sequence of Olupi_PT2024 is marked in red. *cox1* sequences derived from complete mtDNAs, thus corresponding to the entire gene, are reported in bold. The analyzed sequences are listed in Table S1. Sample host and locality are reported only for the *Onchocerca* species. NM, New Mexico; AZ, Arizona; NA, data not available.

The phylogenetic trees reconstructed from both the “longest_cox1” (Fig. 4) and the “barcode_cox1” alignments (not shown) showed the same topology. *O. lupi* and all other analyzed species (i.e., *O. flexuosa, O. ocheng*i, and *O. volvulus*) were monophyletic and highly supported (bootstrap 98%–100%). Moreover, *O. ochengi* and *O. volvulus* were grouped in a well-supported clade (bootstrap 100%). A high bootstrap value (89%, Fig. 4) supported the clustering of *O. lupi* to the clade of *O. volvulus* plus *O. ochengi*. Within *O. lupi*, the *cox1* tree showed the existence of two distinct and highly supported clades: one consisting of “genotype 2,” including Olupi_PT2024 and other samples from the Iberian Peninsula, and the other consisting of “genotype 1,” including specimens from all other parts of the world.

### The mitochondrial genome

**Fig 5:**
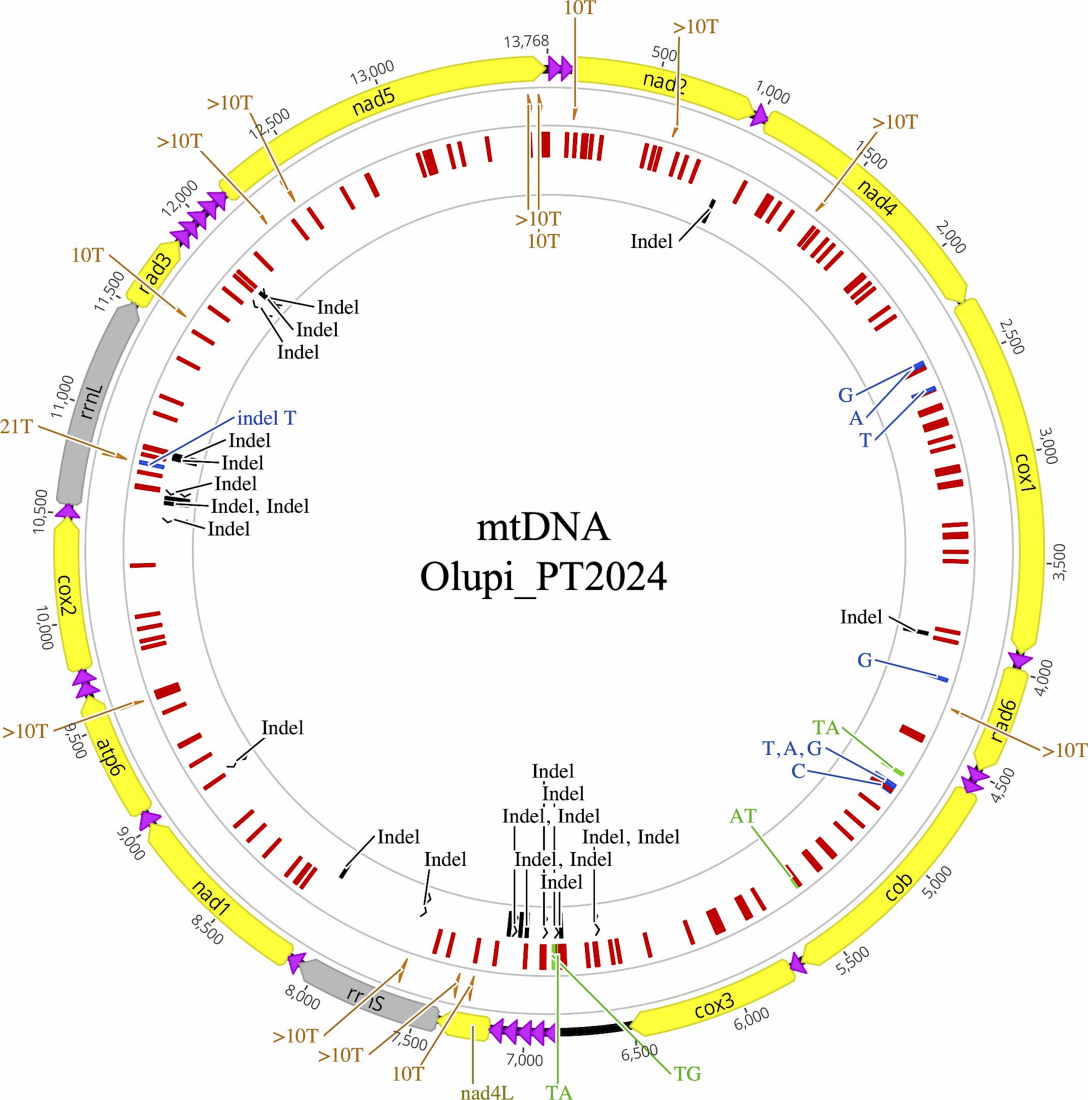
Mitochondrial genome map of the Olupi_PT2024 “genotype 2” (Algarve, Portugal) reporting the 150–154 nucleotide positions differing from the two available mtDNAs of *O. lupi* “genotype 1” (MW266120 and OL964949). Yellow: protein-coding genes; black: *rrnS*, *rrnL* rRNA genes; violet: tRNA genes; orange: A or T omopolymers ≥10 bp. Positions differing from one or both mtDNAs of “genotype 1” are reported in red for single-nucleotide variants (SNVs); light blue for indels; green for variants of length >1 nucleotide. Blue indicates positions differing between the two “genotype 1” mtDNAs. *atp6*: ATPase subunit 6; *cox1–3*: cytochrome oxidase subunits 1 to 3; *nad1–6*, *nad4L*: NADH dehydrogenase subunits 1 to 6 and subunit 4L; *cob*: cytochrome b. tRNA genes are indicated according to the transported amino acid and recognized codons. The genes point in the direction of their transcriptional orientation.

The mtDNA of Olupi*_*PT2024 was identical in size, base composition, gene content, and order to the two genotype 1 mtDNA sequences available, sampled at different times in New Mexico and Arizona (Table S1). Specifically, the Olupi_PT2024 mtDNA was 13,768 bp long, thus 1–2 nt longer than the two genotype 1 mtDNAs. The base composition showed a strong bias toward A+T in all three *O. lupi* mtDNAs (average AT%: 73.43 ± 0.05), with a high number of A and T homopolymers ≥10 bp (in orange in Fig. 5).

The gene content consisted of 22 tRNAs, two rRNAs (large subunit ribosomal RNA, *rrnL,* and small subunit ribosomal RNA, *rrnS*), and 12 protein-coding genes (PCGs; i.e., all canonical mitochondrial protein genes except for *atp8*), with all genes encoded by the same strand. The putative mt regulatory region, corresponding to the l-NCR, was located between *cox3* and *trnA* in both genotypes 2 and 1. In Olupi_PT2024, the l-NCR was 326 bp long, thus 3 bp longer than the l-NCR of genotype 1. The same gene content, gene order, l-NCR position, and AT bias were found in the mtDNAs of other *Onchocerca* and *Dirofilaria*, as well as in most other nematodes (63, 64).

Despite the identical size and structure, the Olupi_PT2024 genotype 2 herein examined differed from one/both genotypes 1 mtDNAs at 186–189 nucleotide positions (150–154 single nucleotide variants—SNV—and 36 indels; Fig. 5), resulting in 1.1% nt divergence. On the contrary, the two genotype 1 mtDNAs differed only at 9 positions (8 SNVs and 1 indel; blue signs in Fig. 5 and Fig. S4), with a divergence of 0.06%. In all comparisons, indels were mostly changes in the length of A and T homopolymeric stretches and were mainly located in *rrnL* and in two tRNA gene clusters (Fig. 5). As for SNVs, they were located only in the *cox1*, *cob,* and *nad6* genes in the comparison within genotype 1 (Fig. S4), while they were distributed throughout all the mt genes, as well as in the longest NCR in the comparison between genotypes (Fig. 5). Excluding the tRNA genes, the highest nucleotide divergence between genotypes was found in l-NCR (3.76%) and *nad4* (2.06%), and the lowest in *nad6* (0.31%) and *rrnS* (0.29%), with the remaining genes showing a divergence in the range of 0.71%–1.41% (average: 1.08% ± 0.24%).

**Fig 6:**
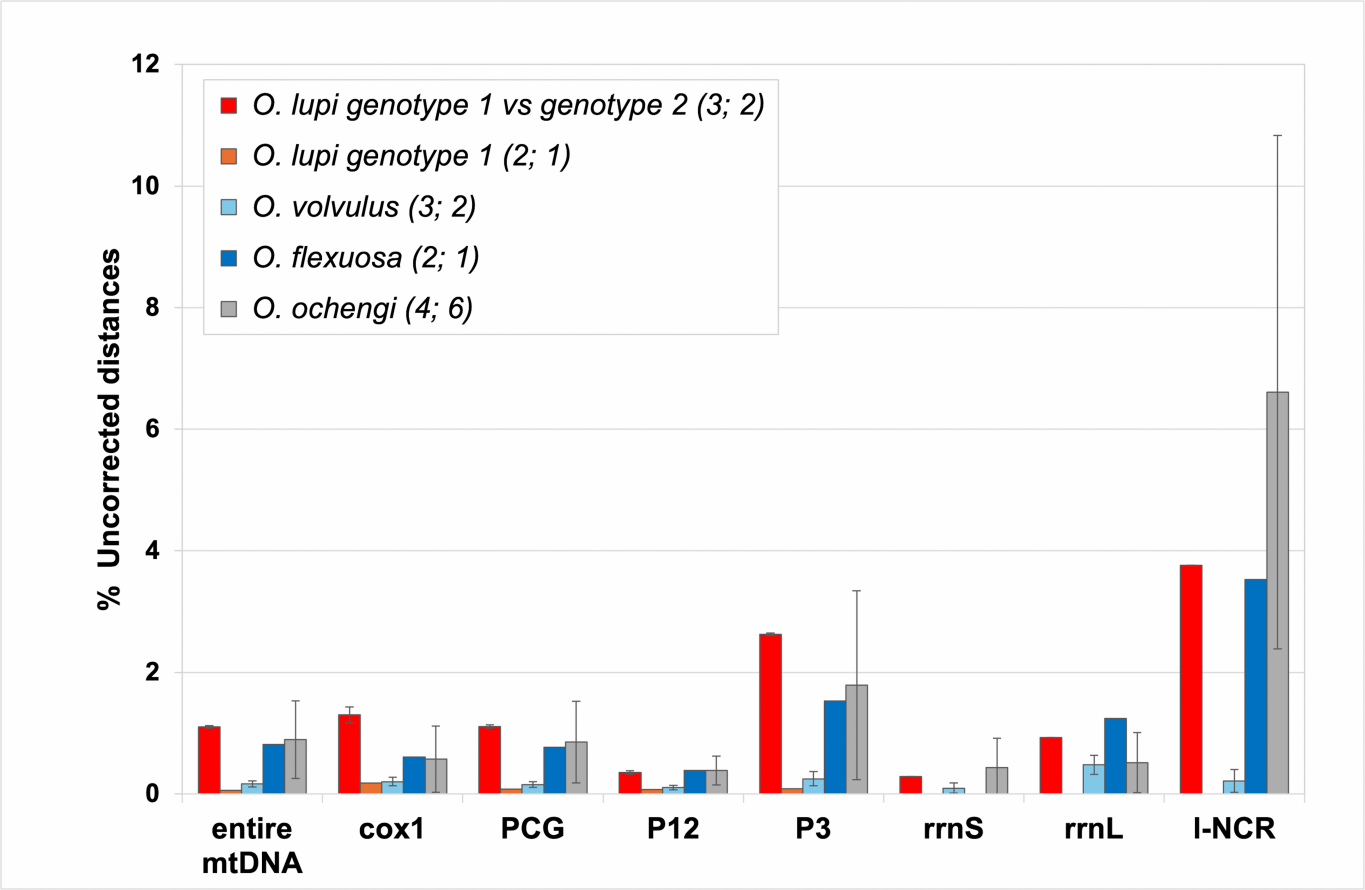
Uncorrected distances (in %) for intra-species comparisons in four *Onchocerca* species. The uncorrected distances were calculated on the various functional regions of the mitochondrial genome. entire mtDNA: entire mitochondrial genome sequence; l-NCR: longest non-coding region; PCG: 12 protein-coding gene; P12: first and second codon positions; P3: third codon position; rrnL: large ribosomal subunit RNA gene; rrnS: small ribosomal subunit RNA gene. In brackets is reported the total number of analyzed mtDNAs and then the number of pairwise comparisons. The mean and standard deviation values are reported in Table S4.

The divergence values between genotype 1 and genotype 2 found on the whole mtDNA and on the various functional regions were consistent with the intra-species divergence found in the three analyzed *Onchocerca* species (Fig. 6 and Table S4).

As expected, l-NCR and P3 were the fast-evolving regions/sites, while P12 was the slowest evolving site. For a given functional region, the intra-species divergence was quite different among species. In all analyzed regions, the divergence within genotype 1 was very low and comparable to the intra-species divergence within *O. volvulus* (orange and light blue bars, respectively, Fig. 6). On the contrary, the divergence between *O. lupi* genotype 2 and genotype 1 was much higher than the divergence within genotype 1 (red vs orange bars in Fig. 6) and closer to the intra-species divergence observed in *O. flexuosa* and *O. ochengi* (red vs blue and gray bars, respectively, in Fig. 6). The *cox1* intra-species divergence closely reflected the divergence found throughout the entire mtDNA. Notably, in all intra-species comparisons, the *rrnS* showed a very low divergence, comparable to P12.

## DISCUSSION

This study provides valuable genomic resources for *O. lupi* by reporting, for the first time, both the nuclear and mitochondrial genomes of an adult specimen isolated from a dog in Portugal. The application of PacBio long-read sequencing technology enabled the reconstruction of the Olupi_PT2024 nuclear assembly, distinguished by high accuracy and completeness. Indeed, when compared with the Olupi_Ro2020_NM assembly of genotype 1, the Olupi_PT2024 assembly exhibits an improved contiguity, i.e., fewer contigs (295 vs 2,320), a higher N50 (670 kb vs 96 kb), and a lower percentage of BUSCO fragmented genes (0.5% vs 1.7%) (Table 1). The data also indicate that the two *O. lupi* specimens possess nuclear genomes nearly identical in size (~92 Gb), as supported by the observation that both assemblies have a high completeness (95% of BUSCO complete single-copy plus duplicate genes, Table 1). However, the absence of gene, repeat, and contaminant annotations in Olupi*_*Ro2020_NM (18) limits further comparative analyses between these genomes.

The distinction between the two *O. lupi* genotypes, initially identified only based on *cox1* and/or *nad5* markers (5, 16, 25), has now been corroborated by the complete mitochondrial genome and other functional mitochondrial regions (Fig. 6). The usefulness of whole mtDNA sequencing for population genetic and phylogeographic investigations, as well as for precisely delineating parasite transmission zones, has been well demonstrated in other nematodes, such as *O. volvulus* and *Thelazia callipaeda* (21, 65–68). For example, the variability observed in the mtDNA of *O. volvulus* mfs has revealed distinct genetic structures corresponding to different geographic areas (67, 69, 70). However, for *O. lupi*, the sequencing and comparison of a large data set of complete mtDNAs of both genotype 1 and genotype 2 is still necessary to fully elucidate the origin, evolutionary history, population genetic structure, and phylogeography of this parasite. The divergence between the two *O. lupi* genotypes, calculated on the whole mtDNA, as well as in the various mitochondrial functional regions, was very similar to the intra-species divergence found in other *Onchocerca* species (Fig. 6), thus both genotypes should belong to the same species. A similar approach, involving intra- and inter-species comparisons, showed that *O. ochengi* and *Onchocerca* sp. “Siisa” belong to the same species (71). However, in this study, only a limited number of intra-species comparisons were available, and the loss of comprehensive metadata for some specimens of *Onchocerca* spp. hindered the determination of their population origins (Table S1).

The few SNVs found within genotype 1 (located in *cox1*, *cob*, and *nad6* genes), together with an almost uniform distribution of SNVs in the 12 PCGs between genotype 1 and genotype 2, confirmed *cox1* as the most effective DNA barcode for *O. lupi*, capable of both species identification and haplotype discrimination.

The low inter-genotype divergence observed in the *rrnS* gene explains the limited resolution of previously published *O. lupi rrnS* phylogenetic trees (25, 72), thus its inadequacy for population genetics and phylogeographic studies. However, it is not excluded that mitochondrial comparative analyses conducted without consideration of the mitochondrial gene boundaries could identify regions suitable for designing additional reliable DNA barcodes.

This study also presents the complete genome of a *Wolbachia* endosymbiont from *O. lupi* genotype 2 (i.e., wb_Olupi_PT2024). Indeed, no *Wolbachia* genome was reported from the *O. lupi* genotype 1 Olupi_Ro2020_NM from New Mexico (18), although the preliminary analyses herein carried out indicated the presence of *Wolbachia*-like sequences in the associated reads (data not shown). Accordingly, other *Onchocerca* species were found to be infected by *Wolbachia*, as evaluated either molecularly or by immune-histochemical methods (30). The only *O. flexuosa* is *Wolbachia*-free, likely due to a secondary loss, as demonstrated by the presence of a large number of endosymbiont pseudogenes in the host nematode nuclear genome (73).

Overall, the phylogenetic tree of *Wolbachia* clustered wb_Olupi_PT2024 with those of the genus *Onchocerca* inside supergroup C (Fig. 3). This supergroup was unique in showing strong genomic hallmarks of long-term host-*Wolbachia* co-evolution (30, 74). Indeed, the genome size of supergroup C was highly compact (0.86–0.99 Mb) and differed from supergroup D (increasing to 1.07–1.1 Mb), which was found in other lymphatic filarioids (e.g., *Wuchereria bancrofti*, *Brugia* spp., Fig. 3). The clustering of the supergroup C within the clade of the supergroup C+J confirmed previous studies where the supergroup J was identified as a basal branch in the C+J clade or as sister group to supergroup C (30, 75, 76), in spite of the limitation in supergroup designation through *wsp* inferences compared to MLST ones (57).

The assembly of the *Wolbachia* entire genome may provide useful targets for anti-onchocercid therapies, via antibiotic-mediated nematode sterilization or killing (77–80). Indeed, the role of *Wolbachia* as a target for treatment against filariods in dogs and humans has been well documented, particularly through doxycycline treatment against *Dirofilaria immitis* (81, 82), as well as *O. volvulus* (34, 77). The comparison of wb_Olupi*_*PT2024 with the *Wolbachia* genome potentially retrievable from the *O. lupi* genotype 1 assembly from New Mexico may reveal genotype-specific differences in gene content or metabolic pathways relevant to the host-symbiont interaction.

### Conclusion

This study provides the nuclear and mitochondrial genomes of an adult *O. lupi* specimen from Portugal, alongside the complete genome of its *Wolbachia* endosymbiont, generated using a shotgun sequencing and *de novo* assembly approach. These genomic data could facilitate the development of novel diagnostic tools useful for refined genetic population studies and thus for delineation of the transmission zone boundaries for each genotype. Furthermore, by integrating genomic and transcriptomic data, non-invasive tests (i.e., serological tests) could be developed for detecting the infection in both adults and mfs. Such an approach could be instrumental in developing policy strategies to improve the diagnosis of onchocerciasis in animals and humans worldwide.

## Data Availability

The authors declare that all data supporting the findings of this study are available within the article and its supplemental files. DNA raw sequencing data and *O. lupi* nuclear, mitochondrial, and *Wolbachia* endosymbiont genome assemblies have been deposited in the ENA database under project accession PRJEB93972.

## SUPPLEMENTAL MATERIAL

Supplemental figures msphere.00625-25-s0001.pdf Figures S1-S4.

Supplemental table captions msphere.00625-25-s0002.pdf Table S1-S4 captions.

Supplemental tables msphere.00625-25-s0003.xlsx Tables S1-S4.

ASM does not own the copyrights to Supplemental Material that may be linked to, or accessed through, an article. The authors have granted ASM a non-exclusive, world-wide license to publish the Supplemental Material files. Please contact the corresponding author directly for reuse.
