# Supplementary material for: Nuclear, mitochondrial, and Wolbachia endosymbiont genomes of Onchocerca lupi, Portugal
Source: mSphere. 2026 Jan 26;11(2):e00625-25. doi: 10.1128/msphere.00625-25 (PMC12931274; doi:10.1128/msphere.00625-25)

Fig S1. Genomic profile of *O.lupi* Olupi_PT2024 obtained from Femto Pulse System with Genomic DNA 165 kb Kit. The Femto Pulse measurement showed a peak at about 8.9 kb. The extracted DNA exhibited no significant smear below the peak size, indicating the absence of degradation. Finally, 56.8% of the fragments resulted between 5-10 kb.

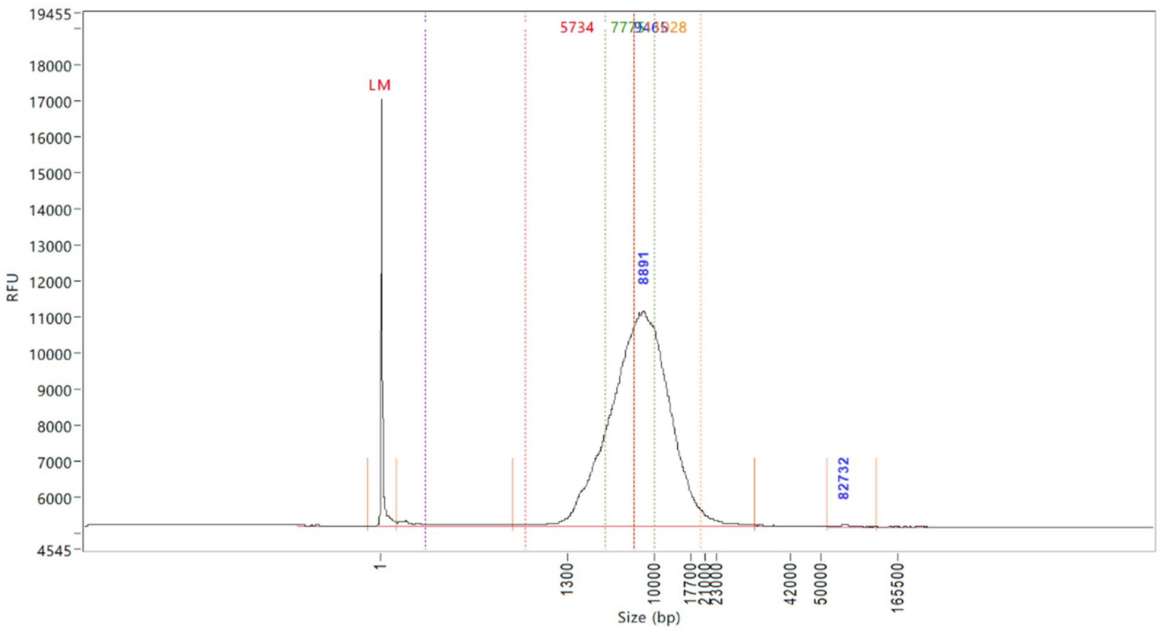

Peak Table

Peak	Size	Concentration	Relative concentration	Molarity	From	To	Average size	Percent CV	RFU	Corrected peak area
	(bp)	(pg/ul)	Percent	(pmole/l)	(bp)	(bp)	(bp)			
1	1 (LM)	1.0175		202.8050	0	111	8	250.22	11875	71.072
2	8891	107.0622	99.8	19.8332	916	32826	8887	44.69	5967	747.826
3	82732	0.1767	0.2	0.0032	60535	132769	90671	15.29	57	1.234
	TIC:	107.2389	Pg/ul							
	TIM:	19.8364	Pmole/l							
	Total concentration:	109.6023	Pg/ul							

Smear Analysis	1000 bp to 8000 bp	47.3579 pg/ul	43.2 %Total	13.5975 pmole/L	5734 Avg. Size (bp)	30.29 %CV
	8000 bp to 20000 bp	58.2918 pg/ul	53.2 %Total	8.7020 pmole/L	11028 Avg. Size (bp)	24.39 %CV
	5000 bp to 10000 bp	62.2476 pg/ul	56.8 %Total	13.1801 pmole/L	7775 Avg. Size (bp)	17.59 %CV
	5000 bp to 20000 bp	91.2267 pg/ul	83.2 %Total	15.8677 pmole/L	9465 Avg. Size (bp)	32.14 %CV

Fig S2. Genomescope2 k-mer profile of Olupi_PT2024.

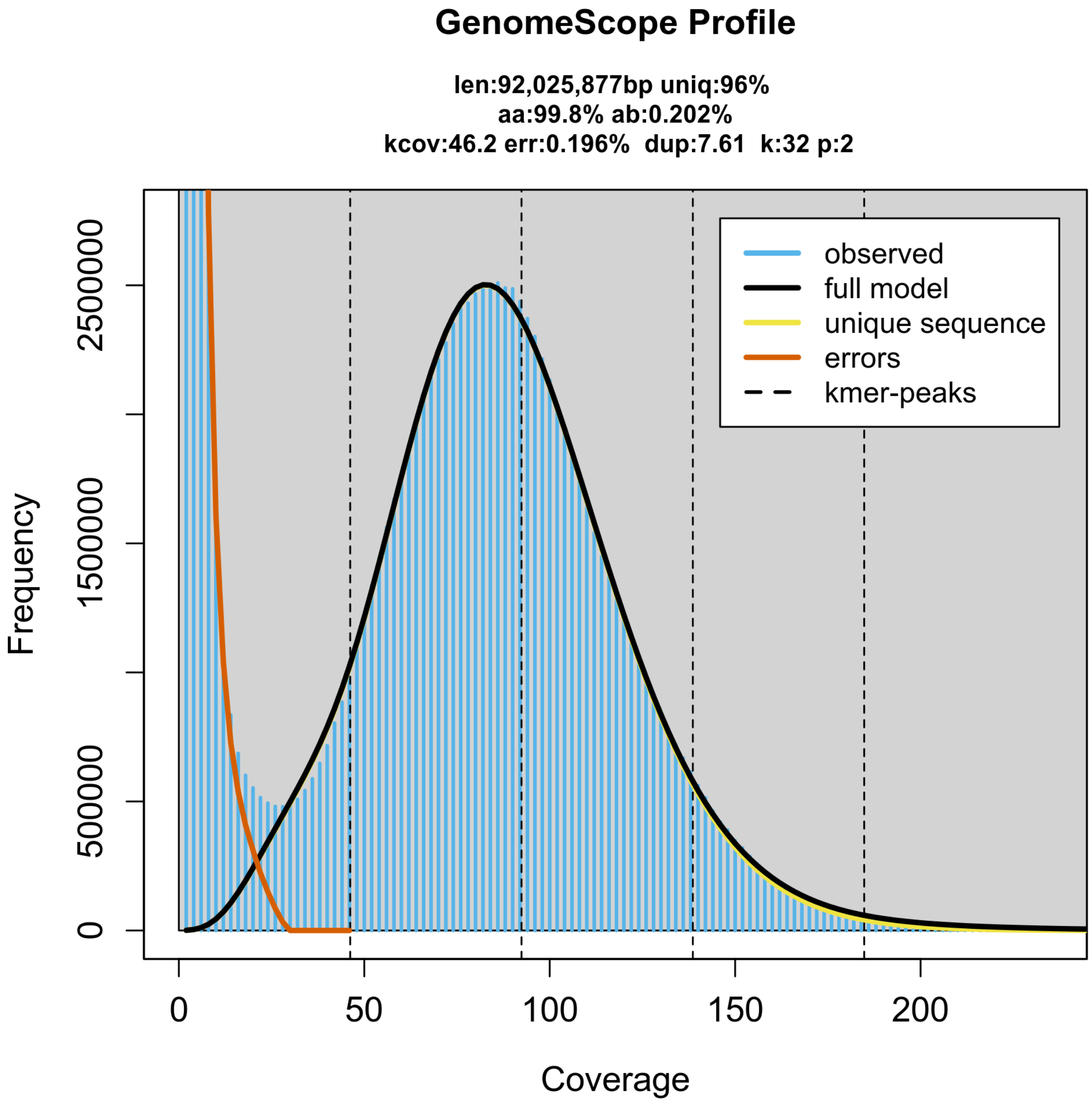

Fig S3. (A) Merqury assembly spectrum plot of Olupi_PT2024 genome. (B) Merqury copy number spectrum plot Olupi_PT2024 genome.

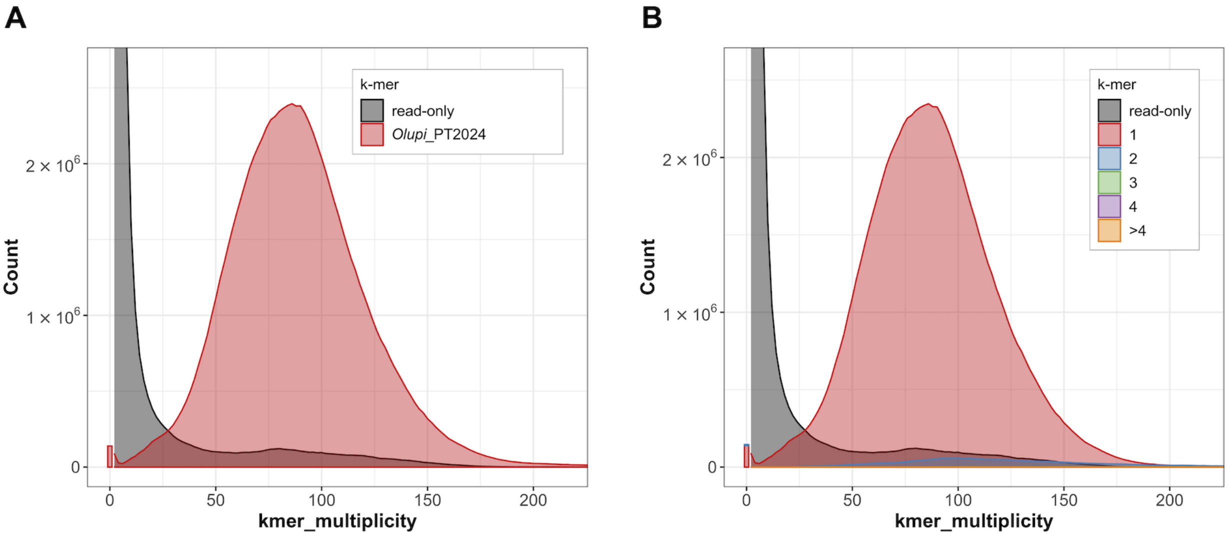

Fig S4. Mitochondrial genome map of the MW266120 O. lupi "genotype 1" (Flagstaff, AZ, USA) reporting the nine nucleotide positions differing from the OL964949 O. lupi "genotype 1" (Albuquerque NM, USA). Yellow: protein-coding genes; black: rrnS, rrnL rRNA genes; violet: tRNA genes; orange: A or T omopolymers ≥ 10 bp. Nucleotide variants present in the Albuquerque OL964949 "genotype 1" are in blue. The genes are pointing in the direction of their transcriptional orientation.

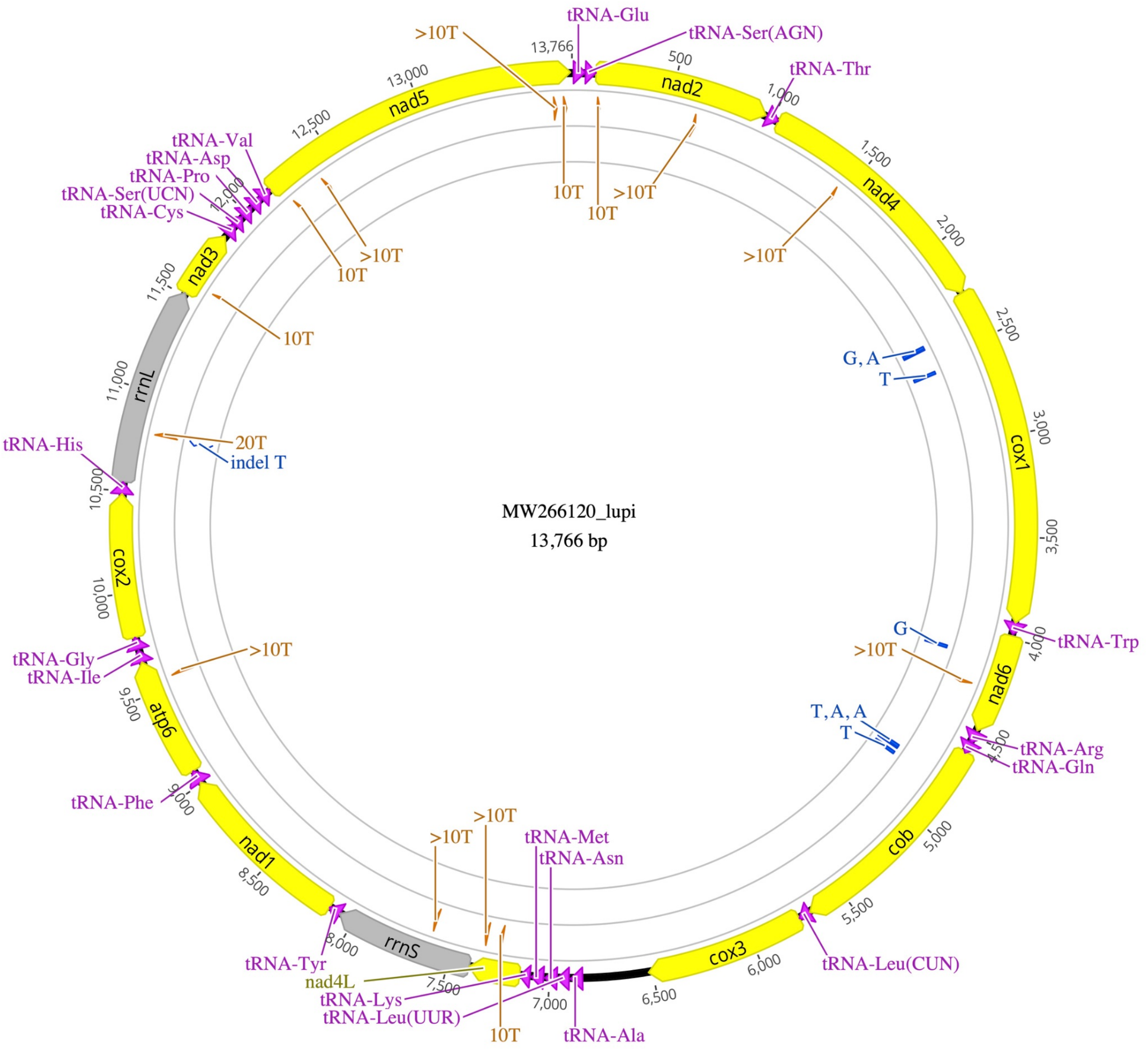

Supplement: Supplemental figures — Figures S1-S4. [file msphere.00625-25-s0001.pdf]
