# Supplementary material for: Nuclear, mitochondrial, and Wolbachia endosymbiont genomes of Onchocerca lupi, Portugal
Source: mSphere. 2026 Jan 26;11(2):e00625-25. doi: 10.1128/msphere.00625-25 (PMC12931274; doi:10.1128/msphere.00625-25)
Supplement: Supplemental table captions — Table S1-S4 captions. [file msphere.00625-25-s0002.pdf]

S1 Table. ENA Accession numbers, together with metadata, of the *cox1* and the complete mtDNA sequences of *Onchocerca* and *Dirofilaria* species analysed in this study. For all sequences is reported entry description, source locality, host, and sequence length. For *Onchocerca* mtDNAs, it is also reported the sampling date. NA: data Not Available

S2 Table. ENA Accession numbers of the *wsp* sequences from seven representative *Wolbachia* (wb) supergroups analysed in this ML tree of Fig 3. For all sequences is reported description, host species and classification, *Wolbachia* supergroup and sequence length. Source locality is reported only for *Onchocerca lupi*. Supergroup assignment is based on MLST literature data.

S3 Table. List of the available *Wolbachia* genomes from Onchocercidae, with the relative length, completeness %, supergroup classification, and NCBI web source. For each genome, the completeness %, obtained using CheckM analysis (v1.2.3), was retrieved from the corresponding "https://www.ncbi.nlm.nih.gov/datasets/genome/" page. Supergroup assignment is based on MLST literature data.

S4 Table. Mean and Standard deviation values of the uncorrected distances (in %) for the intra-species mitochondrial comparisons reported in Fig 6, calculated on the various functional regions of the mitochondrial genome. entire mtDNA: entire mitochondrial genome sequence; l-NCR: longest Non-Coding Region; PCG: 12 Protein-Coding Gene; P12: first and second codon positions; P3: third codon position; *rrnL*: large ribosomal subunit RNA gene; *rrnS*: small ribosomal subunit RNA gene. After the pairwise name, in brackets is reported the total number of analysed mtDNAs and then the number of pairwise comparisons.
